# Unraveling the multiscale structural organization and connectivity of the human brain: the role of diffusion MRI

**DOI:** 10.3389/fnana.2015.00077

**Published:** 2015-06-09

**Authors:** Matteo Bastiani, Alard Roebroeck

**Affiliations:** Department of Cognitive Neuroscience, Faculty of Psychology and Neuroscience, Maastricht UniversityMaastricht, Netherlands

**Keywords:** diffusion MRI, cortical layers and areas, cytoarchitecture, myeloachtecture, structural connectivity

## Abstract

The structural architecture and the anatomical connectivity of the human brain show different organizational principles at distinct spatial scales. Histological staining and light microscopy techniques have been widely used in classical neuroanatomical studies to unravel brain organization. Using such techniques is a laborious task performed on 2-dimensional histological sections by skilled anatomists possibly aided by semi-automated algorithms. With the recent advent of modern magnetic resonance imaging (MRI) contrast mechanisms, cortical layers and columns can now be reliably identified and their structural properties quantified post-mortem. These developments are allowing the investigation of neuroanatomical features of the brain at a spatial resolution that could be interfaced with that of histology. Diffusion MRI and tractography techniques, in particular, have been used to probe the architecture of both white and gray matter in three dimensions. Combined with mathematical network analysis, these techniques are increasingly influential in the investigation of the macro-, meso-, and microscopic organization of brain connectivity and anatomy, both *in vivo* and *ex vivo*. Diffusion MRI-based techniques in combination with histology approaches can therefore support the endeavor of creating multimodal atlases that take into account the different spatial scales or levels on which the brain is organized. The aim of this review is to illustrate and discuss the structural architecture and the anatomical connectivity of the human brain at different spatial scales and how recently developed diffusion MRI techniques can help investigate these.

## Introduction

To fully understand how the brain works in both normal and pathological conditions, we need both functional and anatomical maps. The endeavor of describing the anatomy of the brain has been greatly advanced during the last century. The brain is a complex electrochemical device and, as such, it comprises several fundamental components (i.e., neurons and neuroglia), which are highly interconnected. To reverse engineer any device, we often need to identify its main constituent parts and how these are connected to each other. The same rationale can apply to the study of the nervous system. Therefore, one way to increase our understanding of the brain is to identify its different components and map their afferent and efferent connections.

Importantly, the nature of the components and connections depends on the spatial scale at which the brain is investigated. At the macroscopic level (thousands of microns) the brain is made of different cortical areas and subcortical structures, which are connected to each other via short and long axonal bundles. Zooming in and looking at the mesoscopic organization of the cortical sheet (hundreds of microns), it can be observed that every cortical area shows a different layered organization of cells and neurites and that every layer has its own intracortical connectivity pattern. Finally, from the microscopic viewpoint (tens of microns), axons in white matter are not equally distributed everywhere, but have different packing densities and diameters.

Recent developments in the field of magnetic resonance imaging (MRI) have made it possible to probe both the function and the anatomy of the brain *in vivo* at sub-millimeter resolution. One MRI method in particular is gaining importance for the study of anatomy and structural connectivity of the brain: diffusion magnetic resonance imaging (dMRI). This technique is sensitive to the diffusion of water molecules within the different biological compartments in the brain, such as intracellular and extracellular compartments. This enables it to delineate with high accuracy the areas that have been affected by a stroke as well as to identify the main axonal orientation of neuronal fibers within an imaging voxel. Moreover, although dMRI has been mostly used to probe macroscale brain connectivity within white matter, it has recently also been used to highlight mesoscopic differences in lamination patterns within human cortical gray matter. Finally, dMRI-based techniques have also shown the potential to study the microscopic organization of white matter by estimating axonal packing densities and diameters.

Given these premises, the aim of this work is to review current knowledge about the structural architecture of the brain at different scales and how such knowledge can be advanced using dMRI. Diffusion MRI and dMRI-based techniques are first introduced together with their capability to resolve the anatomical connectivity patterns of the brain. After this, the power of dMRI to probe neuroanatomy at three different scales, namely the macro-, meso-, and microscopic ones, is discussed. This is done by reviewing both some of the most prominent neuroanatomical studies based on histological techniques and recent works coming from the dMRI field. The ultimate aim of this review is to provide the neuroimaging community, which comprises both neuroscientists and clinicians, with a description of tools and evidence that can help to understand the structural architecture of the brain.

## Diffusion MRI

The development of MR neuroimaging techniques has made it possible to reach sub-millimeter resolution to map both the anatomy and the function of the brain. However, standard MRI acquisitions based on T1 and T2 contrasts give only a partial description of the brain’s microstructural composition. Moreover, these contrasts contain little information about the anatomical *connectivity* between different cortical regions. Diffusion MRI (dMRI) overcomes some of these limitations. Diffusion MRI encodes the displacement of water molecules within the different brain compartments, which can be used to characterize the brain’s structural organization.

### Physical and Imaging Principles of Diffusion MRI

Free-floating molecules in a gaseous or fluid medium display thermal motion in a random walk pattern: a behavior known as Brownian motion or passive diffusion. Einstein (1905) formalized this diffusion behavior in mathematical terms for randomly diffusing particles in an isotropic medium (i.e., free diffusion). Statistically, the displacement probability distribution within an isotropic medium can be modeled using a Gaussian distribution, with its variance and isoprobability contours moving outward with time. However, in the brain, cell membranes, organelles, and myelin sheaths create barriers and form biological compartments that constrain the displacement of water molecules, modifying their statistical behavior over time. Water diffusion measured over all but the shortest time intervals in biological tissue such as the brain is typically hindered or restricted by these barriers and anisotropic in nature. Measuring water diffusion with dMRI characterizes the so-called apparent diffusion coefficient (ADC) dependent on physical factors (diffusing molecule and viscosity of the medium), parameters of the measurement (discussed below) and the biophysical environment of barriers and compartments. Brain white matter mostly consists of myelinated axons that are often reasonably coherently organized in bundles or tracts. There are two important microstructure compartments in white matter in which anisotropic water diffusion can be measured with dMRI: the intra–axonal and an extra–axonal compartment. Water diffusion within the confines of the axonal membrane is said to be restricted, while diffusion within the extracellular space is said to be hindered. The latter can be well approximated using an anisotropic Gaussian probability distribution, but for restricted diffusion the Gaussian assumption does not hold.

**FIGURE 1:**
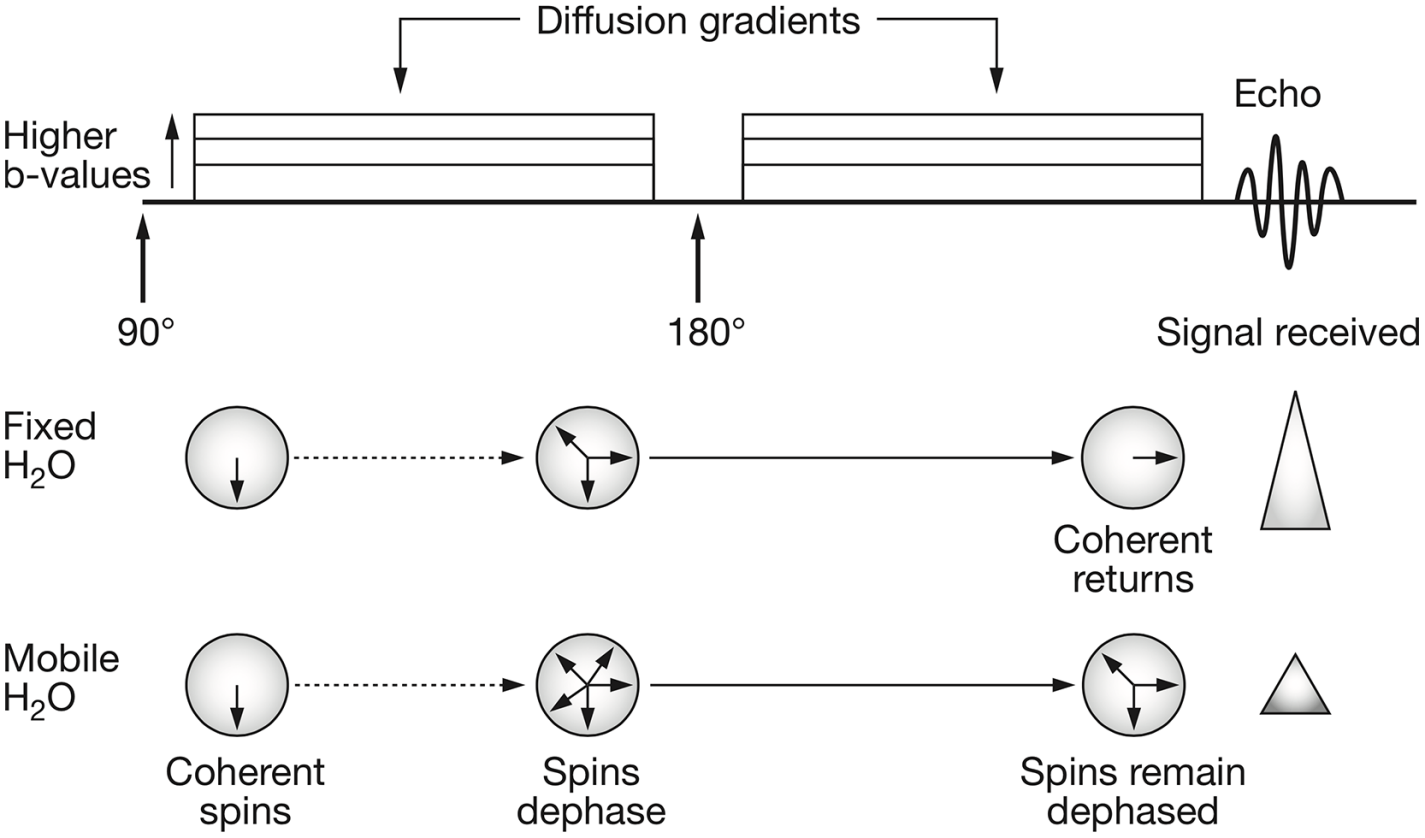
**Schematic representation of the pulsed gradient spin echo (PGSE) diffusion sequence and water spins behavior.** The classical PGSE sequence consists in the application of an initial 90° radiofrequency (RF) pulse and a refocusing 180° RF pulse. When interested in probing the diffusion behavior of water molecules, two diffusion gradient pulses are applied. These are characterized by amplitude (|G|), duration (δ) and time between their application (Δ). The diffusion gradients tag the spins of moving water molecules. This means that, those spins that move along the direction of the applied q-vector will elicit a signal reduction since they will have lost their coherence. The free induction decay signal is then acquired at echo time. Reprinted by permission from Macmillan Publishers Ltd (Patterson et al., 2008).

To measure the displacement of moving spins with dMRI, the pulsed gradient spin echo (PGSE) sequence is mostly used (Stejskal and Tanner, 1965). In this MRI pulse sequence, two high-amplitude diffusion gradients are applied to dephase and, after a spin-echo refocusing pulse has been delivered, rephase stationary spins (protons, **Figure 1**). Spins that are moving along the direction of the applied diffusion gradient will show bulk dephasing and, consequently, signal loss. The signal loss is proportional to the amount of diffusion, the area under the diffusion encoding gradients and to the time interval between the two gradients. In a mono-exponential signal decay model, the *b*-value is used as a measure of diffusion encoding in the diffusion gradients:

(1) $$b=\gamma^{2}\delta^{2}|G{|}^{2}\left(\Delta -\frac{\delta }{3}\right)$$

where *b* is the *b*-value measured in s/mm^2^, γ is the gyromagnetic ratio, δ is the duration of the diffusion pulse, | G| is the amplitude of the diffusion gradients and Δ is the time interval between the two diffusion gradients (**Figure 1**). The monoexponential approximation is valid at low *b*-values and models the signal as:

(2) $$S_{i}=S_{0}e^{-b\;D_{i}}$$

where *S*_i_ is the measured signal with the *i*^th^ diffusion encoding gradient in direction 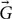
_i_= [x_i_, y_i_, z_i_], S_0_ is the measured signal without diffusion encoding, *b* is the *b*-value and *D*_i_ is the ADC. A dMRI experiment will consist of a few unweighted *S*_0_ measurements and a larger number of measurements with different diffusion gradient directions. Under the narrow-pulse approximation (δ<<Δ), the ADC *D*_i_ can then be estimated in every voxel of the image and for every sampled diffusion direction 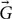
_i_

**FIGURE 2:**
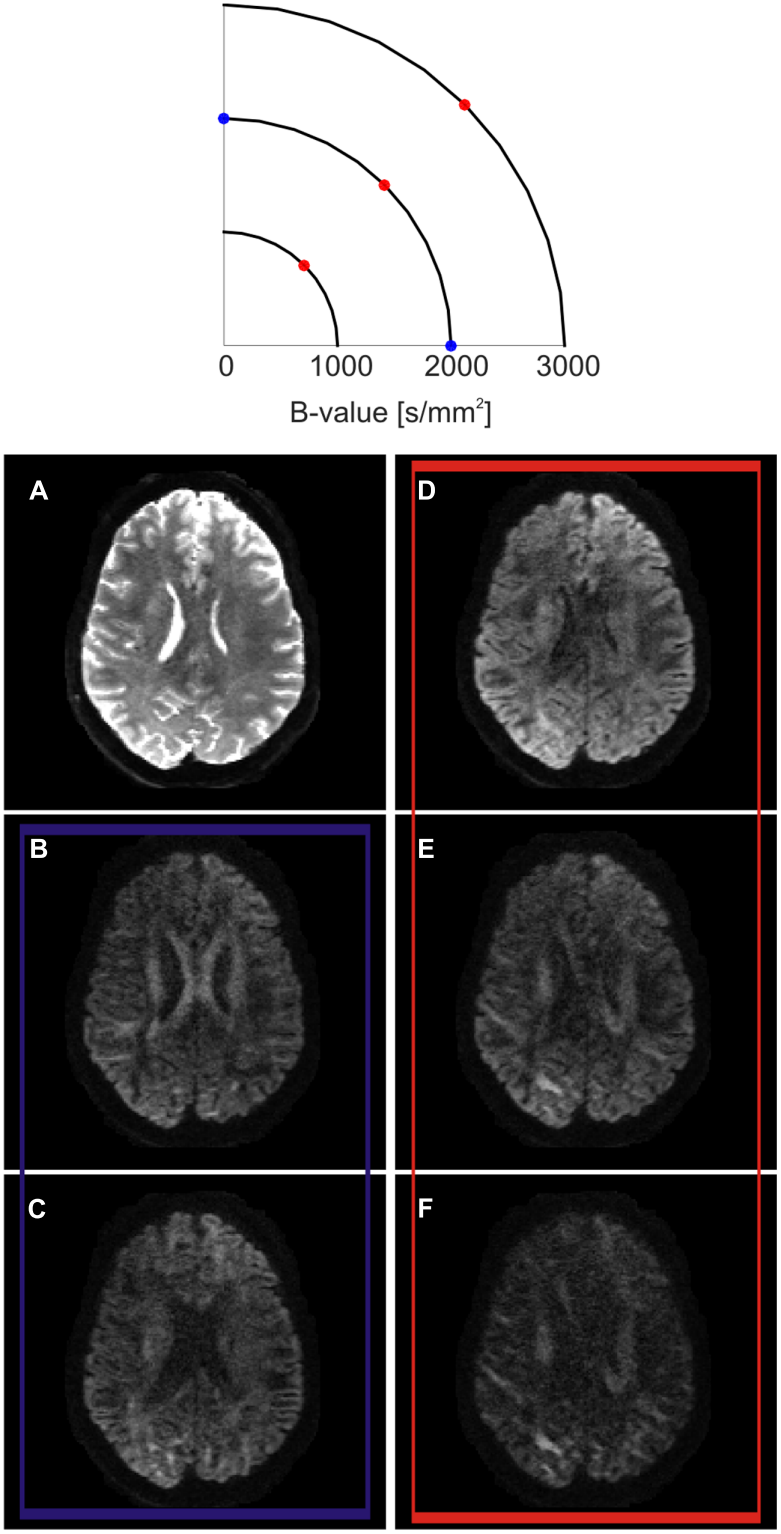
**Q-space encoding example.** Q-space is represented as a two dimensional space for simplification purposes (top). Three different shells at three different *b*-values are outlined. On each shell, several diffusion encoding directions (q-vectors) are sampled. Example axial slice obtained at different positions in q-space (bottom). **(A)** When the diffusion encoding gradients are switched off, the so-called T2-weighted b0 volume is obtained. **(B,C)** show the effect of sampling q-space at two different diffusion encoding orientations lying on the same b-shell. While SNR and amount of probed diffusion are kept constant, the contrast changes at different anatomical locations. When sampling along the left–right direction, the body of the corpus callosum show a higher signal drop **(C)** in respect to the sampling along the anterior-posterior direction **(B)**. This reflects the fact that, in the body of the corpus callosum, axons are mainly oriented along the left–right orientation. **(D–F)** show the effects of sampling along the same diffusion encoding orientation while increasing *b*-value. In this case, the total SNR decreases while moving from lower to higher *b*-values, but the contrast increases. As an effect, different anatomical structures are more strongly differentiable between each other according to their main axonal orientation.

The diffusion gradients could be said to probe a three-dimensional space called q-space (**Figure 2**; Callaghan et al., 1988; Hagmann et al., 2006). Every sampled point can be represented using vector 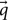:

(3) $$\vec{q}=\frac{\gamma \delta \vec{G}}{2\pi }$$

where 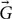 is the diffusion gradient vector. Q-space imaging consists in estimating the full probability distribution of water molecules’ displacement, the so-called diffusion propagator, or its statistical moments, by sampling points in q-space and using the Fourier transform relationship between q-space and the diffusion propagator (Callaghan et al., 1988). Diffusion spectrum imaging (DSI; Wedeen et al., 2005) links the diffusion signal directly to the diffusion propagator by sampling the entire three dimensional q-space on a Cartesian grid and using discrete Fourier analysis.

### Diffusion Tensor Imaging and Beyond

Diffusion tensor imaging (DTI; Basser et al., 1994; Basser and Pierpaoli, 1996) was introduced to characterize diffusion in an anisotropic medium, such as brain white matter with a relatively simple parametric model. DTI today still represents a clinical and research standard for analyzing dMRI data. By sampling q-space using at least six linearly independent diffusion encoding directions and a non-diffusion weighted volume and extending Eq. 2, it is possible to model the diffusion behavior of water molecules in three dimensions using a rank-2 tensor representation:

(4) $$D=\left[\begin{array}{l} D_{xx}\;D_{xy}\;D_{xz}\\ D_{xy}\;D_{yy}\;D_{yz}\\ D_{xz}\;D_{yz}\;D_{zz} \end{array}\right]$$

This symmetric matrix contains six independent components that summarize the diffusion properties of water molecules within a voxel.

**FIGURE 3:**
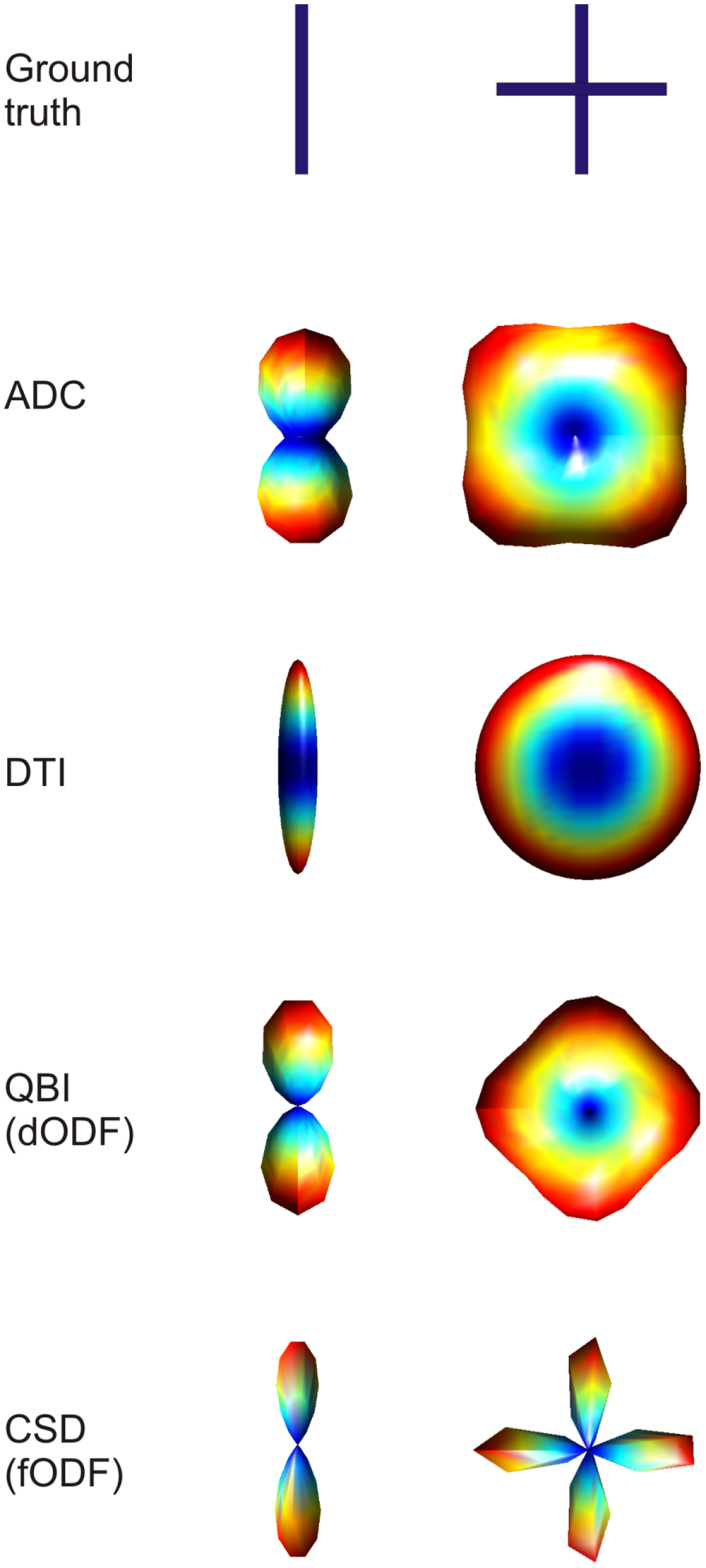
**Example of three-dimensional profiles obtained using different dMRI methods in the case of single fiber configuration and two fibers crossing at 90° (top row).** Visualizing the three-dimensional ADC profile, it can be seen that in the case of crossing fibers, the maxima do not coincide with the true fiber orientations. When using diffusion tensor imaging (DTI), the single fiber case is reconstructed accurately, with the ellipsoid being very anisotropic in the direction of the fiber. When two fibers are crossing the DTI derived ellipsoid will turn into a disc, losing the possibility to discriminate the two main orientations. The last two techniques shown, namely QBI and CSD, reconstruct the three dimensional orientation distribution functions more accurately. The figure shows that the dODF looks less sharply defined when compared to the fODF.

DTI models water diffusion using a Gaussian distribution, i.e., the average displacement due to diffusion of the sampled molecules will be characterized by only one peak. Therefore, using DTI it is only possible to reconstruct one main direction of diffusion. This direction can be found by decomposing (i.e., diagonalizing) the tensorial matrix into so-called eigenvalues and eigenvectors:

(5) $$D={\left[\begin{array}{l} e_{1x}\;e_{2x}\;e_{3x}\\ e_{1y}\;e_{2y}\;e_{3y}\\ e_{1z}\;e_{2z}\;e_{3z} \end{array}\right]}^{T}\cdot \;\left[\begin{array}{l} \lambda_{1}\;0\;0\\ 0\;\lambda_{2}\;0\\ 0\;0\;\lambda_{3} \end{array}\right]\;\cdot \;\left[\begin{array}{l} e_{1x}\;e_{2x}\;e_{3x}\\ e_{1y}\;e_{2y}\;e_{3y}\\ e_{1z}\;e_{2z}\;e_{3z} \end{array}\right]$$

where *e*_1_, *e*_2_, and *e*_3_ are the eigenvectors, λ_1_, λ_2_, and λ_3_ are the eigenvalues and T the transpose operator. Intuitively, this decomposition reflects the amount of displacement due to diffusion (the eigenvalues) along three orthogonal orientations (the eigenvectors). This means that in a highly isotropic voxel (e.g., one that lies within the ventricles) the eigenvalues will roughly be the same and have a relatively high value. On the other hand, in anisotropic voxels (e.g., those lying in the corpus callosum) the eigenvalues will be different from each other, with the largest eigenvalue (usually taken, by reordering, to be λ_1_ and called major eigenvalue) much larger than the other two. The corresponding major eigenvector e_1_ then represents the main direction of diffusion. The complete eigenvalue decomposition allows the representation of the water molecules’ displacement within a voxel as a three-dimensional equiprobability surface, the well-known diffusion tensor ellipsoid (**Figure 3**).

It can be assumed that the main orientation *e*_1_ of this ellipsoid will correspond to the orientation of the axons in those voxels with simple geometry and a high degree of coherence of axonal bundles. Furthermore, several scalar indices can be estimated from the diffusion tensor. Mean diffusivity (MD) measures the average degree of water diffusion within a voxel and is computed as the average of the three eigenvalues. For low *b*-values (i.e., <3000 s/mm^2^), MD does not clearly highlight differences between white and gray matter, but it is very useful to discriminate pathological diffusion behavior due to, e.g., ischemic stroke. Fractional anisotropy (FA; Basser and Pierpaoli, 1996) measures the directional specificity of diffusion as obtained by DTI analysis. FA values, essentially a normalized measure of variance of the eigenvalues, range between 0 (fully isotropic) and 1 (fully anisotropic). Both the MD and FA indices have been widely used to study neurodevelopment (e.g., McKinstry et al., 2002), axonal integrity (e.g., Khayal et al., 2011), clinical conditions (for a review, see: Dong et al., 2004) and plasticity (for a review, see: Zatorre et al., 2012).

As already hinted to, the main limitation of DTI is its incapability of resolving complex fiber architecture within an imaging voxel such as large curvature, divergence or splaying, or two or more differently oriented fibers. Recently it has been shown that at the nominal resolution of current day standard dMRI acquisition (2–3 mm isotropic voxels), more than 90% of voxels contains more than one fiber bundle (Jeurissen et al., 2013). Therefore, more sophisticated models for dMRI data have been proposed that can better represent such complex fiber architecture. In the case of multiple fiber components with different orientations the peaks of the ADC profile do not directly correspond to actual fiber orientations (**Figure 3**). In the case of a 90° crossing, for example, there is an offset of 45° (Alexander et al., 2002; Frank, 2002). Furthermore, the ADC is computed under the assumption of monoexponential signal decay, which breaks down at high *b*-values. Therefore, more sophisticated models for complex fiber architecture tend to avoid the ADC formulation. DSI, (Wedeen et al., 2005) reconstructs the diffusion propagator or displacement probability in a model-free or non-parametric way by directly computing the Fourier transform of the measured q-space signal as defined above. In order for this approach to work it is necessary to sample the q-space very densely (i.e., use a high number of diffusion encoding directions at different *b*-values). A simplification to this model is represented by high angular resolution diffusion imaging (HARDI) acquisitions. By probing a high number of diffusion encoding directions only on one spherical shell in q-space (i.e., using one *b*-value), it is possible to infer non-Gaussian effects in the reconstruction of ADC profiles (Alexander et al., 2002; Frank, 2002). Q-ball imaging (QBI; Tuch, 2004) was the first attempt to model multiple fiber components in a voxel. Using a model-free approach, QBI reconstructs the spin displacement orientation distribution function (dODF). Like the diffusion tensor ellipsoid, the dODF represents a probability function defined over a spherical surface that maps the likelihood of water molecules to move along a certain orientation (in either direction) due to diffusion in three dimensions (**Figure 3**). The dODF peaks can then be assumed to represent the orientations of multiple fiber bundles within a specific voxel. By mapping the water displacement, QBI dODFs are not very sharp as water will still have some directional components orthogonal to the main axon directions. Spherical deconvolution (Tournier et al., 2004) and, subsequently, constrained spherical deconvolution (Tournier et al., 2007), aims at modeling the actual fiber orientation distribution function (fODF), rather than water displacement. The fODF reflects the actual orientations of distinct fiber populations by modeling complex fiber configurations as linear combinations of Dirac’s delta functions (**Figure 3**). Further model-free approaches are such as the diffusion orientation transform (Ozarslan et al., 2006) and persistent angular structure MRI (Jansons and Alexander, 2003; Parker and Alexander, 2005) can be considered as extensions of QBI and spherical deconvolution respectively.

Moreover, a generalization of single shell (i.e., single *b*-value) HARDI-based techniques has been proposed. Generalized q-sampling imaging (GQI; Yeh et al., 2010) increases the sensitivity to multiple fiber compartments which are characterized by different microstructural properties by sampling q-space using multiple shells. Model-based approaches that are capable of resolving more than one fiber population in a voxel have also been proposed. The multi-tensor model is a generalization of the classical DTI approach, where two or more diffusion tensors are fitted to the data (Tuch et al., 2002). A Bayesian framework has been described by which it is possible to fit an isotropic component and several fiber components to the sampled diffusion signal, with an estimate of orientational uncertainty (Behrens et al., 2007). Model-based multi-compartment approaches (Assaf and Basser, 2005; Zhang et al., 2012) have recently been used to probe the microstructural organization of white matter by estimating axonal packing density or neurite dispersion.

### Tractography

Any set of modeled local orientations can be used for tractography or fiber tracking. That is, once the main orientation has been estimated in every voxel, it is possible to reconstruct tracts connecting different brain regions by further modeling techniques. Several algorithms have been developed to perform tractography. These algorithms can be clustered in different classes from the methodological point of view. Those approaches that, given a seed point where the tracking begins, proceed in discrete steps are named streamline or local integration algorithms. These further comprise two sub-classes, deterministic and probabilistic, according to whether they are taking into account the uncertainty in the estimation of the local fiber orientation. Global tractography algorithms, on the other hand, try to optimize the whole set of estimated local orientations at once (Bastiani et al., 2012).

**FIGURE 4:**
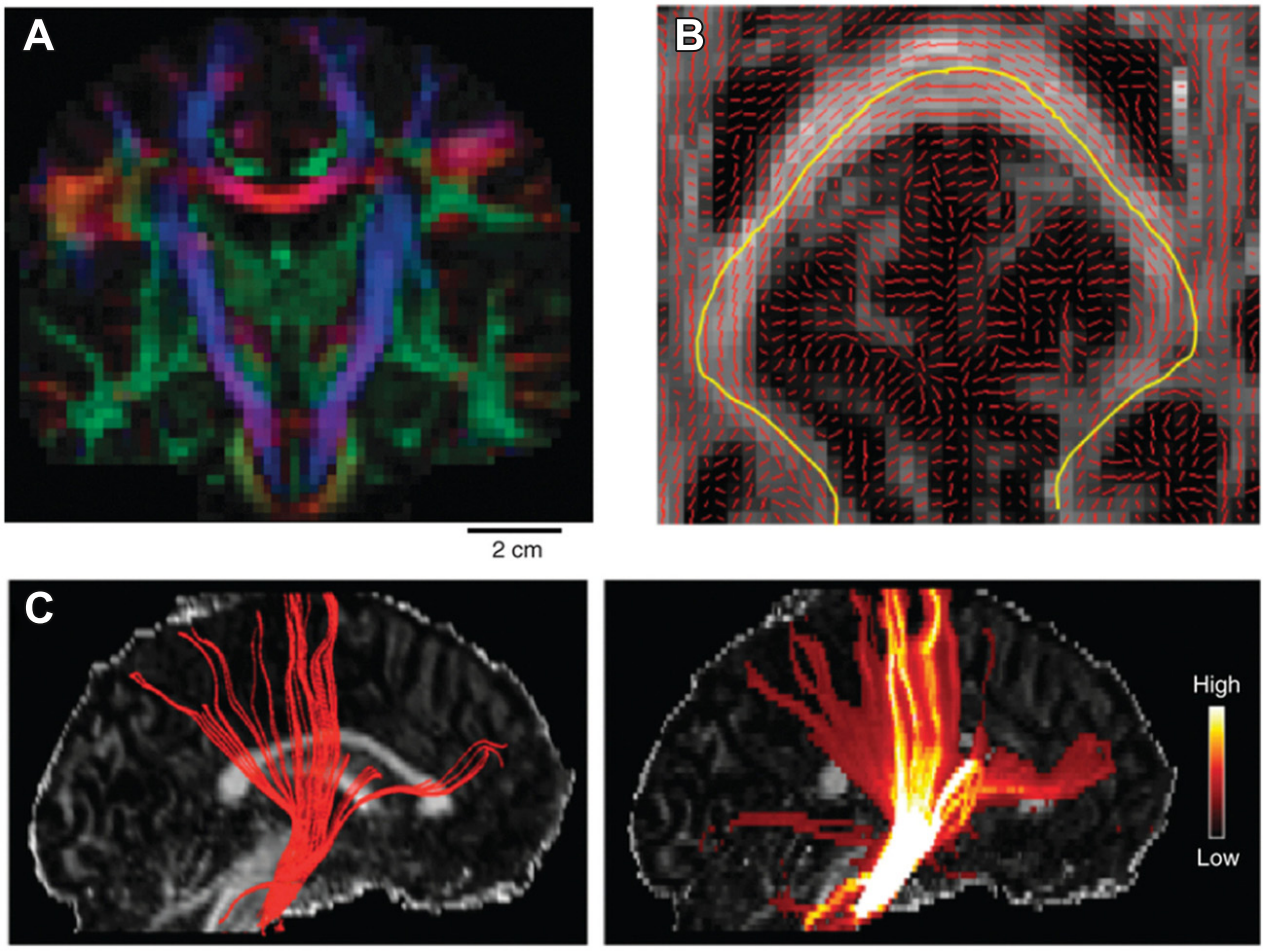
**Example of tractography algorithms. (A)** shows the main DTI eigenvector orientation as a RGB color coded map of a single coronal slice of the human brain. This is obtained by separately mapping the *x, y*, and *z* component of the main eigenvector on the three separate color channels of the RGB color scale (scale bar: 2 cm). **(B)** shows the main eigenvector orientation visualized on top of a FA axial slice. An example streamline is mapped in yellow, which follows the main path crossing the corpus callosum. **(C)** shows the results of tracking the pyramidal tract using a deterministic (left) and a probabilistic (right) approach superimposed on a FA sagittal slice. Color bar on the right indicates the confidence estimate obtained when using probabilistic approaches. Reprinted by permission from Macmillan Publishers Ltd (Craddock et al., 2013).

The classic local deterministic approach was based on DTI and uses a streamline algorithm, which locally integrates the fiber path using a step-wise approach (**Figure 4**). A streamline is initiated at a certain seed point and follows the main eigenvector of the diffusion tensor, switching orientation as soon as it has entered a new voxel (Conturo et al., 1999; Mori et al., 1999; Mori and van Zijl, 2002). Since the paths reconstructed using this method are biased by the coarse spatial resolution of dMRI data, continuous approximation techniques of the tensor field have been proposed to smooth the results and make them follow more biologically plausible pathways (Basser et al., 2000). Despite their simplicity, local deterministic tractography methods have been successfully used to perform *in vivo* ‘virtual dissection’ of known fibers analogous to classical post-mortem fiber dissection techniques (Catani et al., 2002). Using prior knowledge, two regions of interest (ROIs) are selected through which the tractography streamlines should run. The problem of false positive fibers can be partially addressed by selecting a third exclusion ROI to remove the known spurious fibers.

However, dMRI data are inherently corrupted by a certain amount noise, which depends on factors such as voxel size and amount of diffusion weighting (i.e., *b*-value). As a result, the estimation of the diffusion tensor contains a certain amount of uncertainty, which is then reflected in its eigenvalues and eigenvectors (Jones, 2003). Probabilistic tractography approaches characterize the variability of tractography results resulting from the uncertainty of the estimated local fiber directions (Behrens et al., 2003b; Parker et al., 2003). This contrasts them with deterministic approaches which give a single deterministic answer for the connection of a given region-of-interest (ROI) to any part of the brain. In a probabilistic algorithm the direction of track propagation will be randomly selected at every step by sampling the local orientation distribution function. Probabilistic algorithms, therefore, will propagate several hundreds of streamlines from the same seed point and each iteration will result in a slightly different preferential path (**Figure 4**). The result of a probabilistic tractography algorithm for every single well-defined seed point is a three-dimensional map of visitation counts for fibers through a voxel. The noisy streamline or diffusing particle principles have been applied both to tensor models and to complex local architecture models (Parker and Alexander, 2005; Behrens et al., 2007; Tournier et al., 2010).

Local streamline methods, either deterministic, or probabilistic, use only local information to determine the course of tracts. Global approaches move from local step-wise reconstruction of fiber trajectories to a global goodness-of-fit of the entire candidate fiber. Here, the measure of fit quantifies the joint likelihood of the fiber given all voxel data it passes through (Tuch et al., 2002; Jbabdi et al., 2007; Sherbondy et al., 2008, 2009; Zalesky and Fornito, 2009). The global fit measure makes tractography less sensitive to modeling errors caused by local noise (Jbabdi et al., 2007). A graph-based global tractography algorithm (Iturria-Medina et al., 2007) and its extension to a multiple direction fiber models (Sotiropoulos et al., 2010) have been proposed. These algorithms reconceptualize the global tractography problem as a shortest-path search in a graph, in which (in contrast with the connectome graph, described below) nodes are represented by the center of each white-matter voxel. Since graph-weights are then defined as the probability of voxel-center connections given the local ODFs, a shortest path from one point to another in this graph constitutes a globally optimized fiber. Since in a shortest-path search all possible nodes are visited and the path lengths recorded, the n% shortest paths then correspond to the n% most likely paths in the probabilistic tractography sense. Thus, graph-based methods – and in fact, global methods in general – are naturally used as probabilistic methods. The only way to force a global graph based approach to be deterministic is to select only its highest percentile results for consideration, which corresponds to looking at only the single shortest path that connects two points. Some global tractography approaches even aim at constructing the entire connectivity pattern between all the voxels in the acquired volume with minimum user input and interaction. One way toward this that has proven successful is to fit thousands of line segments in the entire sampled volume and optimizing both local fit of the segment to data they pass through and smoothness of the segments (Kreher et al., 2008; Reisert et al., 2011). Global approaches are computationally very expensive, but being less dependent on user choices and more resistant to noise make them very interesting.

## Macroscopic Brain Organization: Cortical Cartography and Connectomics

The cerebral cortex can be subdivided into two major parts, the isocortex and allocortex (Vogt, 1910). The first one shows a clearly defined six-layered structure when looking at Nissl-stained sections in almost all of its parts, while the latter shows wide variability in its microstructural anatomical patterns. More fine-grained parcellations of the whole cortical mantle were first published at the beginning of the 20th century (Brodmann, 1909; Vogt, 1910). Cytoarchitectural parcellations of the cortex have been brought forward by several other groups (von Economo and Koskinas, 1925; Bonin and Bailey, 1947; Sanides, 1962; Zilles and Amunts, 2010, Amunts et al., 2013), while only few studies have focused on myeloarchitecture (for a review, see: Nieuwenhuys, 2013). Recently, new methods have been introduced that can provide details about other structural features of the cortex, such as receptor mapping techniques (Geyer et al., 1998; Eickhoff et al., 2008; Zilles and Amunts, 2009, Amunts et al., 2010).

Whereas neural cell bodies and intracortical neurites form the basis of gray matter architecture, long and short association tracts are the neuroanatomical substrate of white matter. The latter constitutes 40% of the total matter volume in the central nervous system of the adult human brain (Morell, 1984). Theodor Hermann Meynert originally postulated the three principal types of white matter tracts, now well known and accepted: association tracts that link the different brain regions within the same hemisphere, including both short (the U-shaped fibers of Meynert) and long association fibers, commissural tracts that connect the two hemispheres and afferent and efferent projection tracts between the cerebral cortex and subcortical structures.

To study the brain’s structural connectivity profile, several animal studies have used tract tracing techniques that are based on the axonal transport of injected neuronal tracers, either anterogradely, or retrogradely. These studies have allowed the detailed study of cortical networks, such as the hierarchical organization of the visual system in the monkey brain (Felleman and Van Essen, 1991). In most tract tracing studies to date the strength of the connection (the number of projecting axons) was not well quantified. The addition of a weighting index, together with the direction of the connection, represents a fundamental step to unravel the hierarchical structure between functionally specialized brain areas (Markov and Kennedy, 2013). Further studies have developed databases of weighted regional connectivity indices, encoding connection strength, based on tracing studies in the whole monkey cortex (Stephan et al., 2000; Markov et al., 2011; Bakker et al., 2012). Furthermore, since interspecies studies represent a fundamental step to understand brain evolution and development, a translation of these connectivity profiles to the human brain has been attempted, based on macroanatomical landmarks and structural similarities between human and macaques (Kotter and Wanke, 2005; Bezgin et al., 2012).

The neuroscientific endeavor of mapping the whole brain connectivity map represents a fundamental effort to understand cognition (Bressler and Menon, 2010; Van Essen and Ugurbil, 2012). Several attempts to map the macroscopic structural connectivity pattern of the whole brain have been made through history using different techniques (see for review: Catani et al., 2013). To summarize all the results obtained by using different methods in a convenient way, the brain can be conceptualized as a set of nodes (cortical regions and subcortical nuclei) connected by different edges (the axons). Mathematically, a set of nodes and edges is called a graph and an entire branch of mathematics (graph theory) is dedicated to studying it. The graph representing all the cortico-cortical and cortico-subcortical connections between cortical areas and subcortical nuclei has been recently called the (macroscopic) connectome (Sporns et al., 2005).

Diffusion MRI based tractography has made it possible to trace *in vivo* the three principal types of macroscopic white matter tracts that were originally described by Meynert using post-mortem samples. Both the long association tracts and the commissural fibers have been described in great detail in term of their anatomical location and fiber termination area *in vivo* and non-invasively (Wakana et al., 2004; Catani and Thiebaut de Schotten, 2008). Fibers obtained from tractography analysis connecting cortical areas to subcortical structures such as the thalamus have been traced with a level of detail that resemble that of classical post-mortem histological studies (Behrens et al., 2003a). Moreover, advancements in dMRI acquisition techniques and MRI hardware have increased the achievable voxel resolution, allowing short-association tracts (u-fibers) to be identified and segregated in the frontal lobe (Catani et al., 2012). A recent study has used *in vivo* dMRI and post-mortem polarized light imaging (PLI; Axer and Keyserlingk, 2000; Axer et al., 2001, 2011a,b; Larsen et al., 2007; Dammers et al., 2010, 2012; Kleiner et al., 2012) to locate and validate cortical insertion sites of transcallosal fibers in visual cortices in humans, which are mostly located at the boundaries between different cytoarchitectonically defined visual areas (Caspers et al., 2015).

**FIGURE 5:**
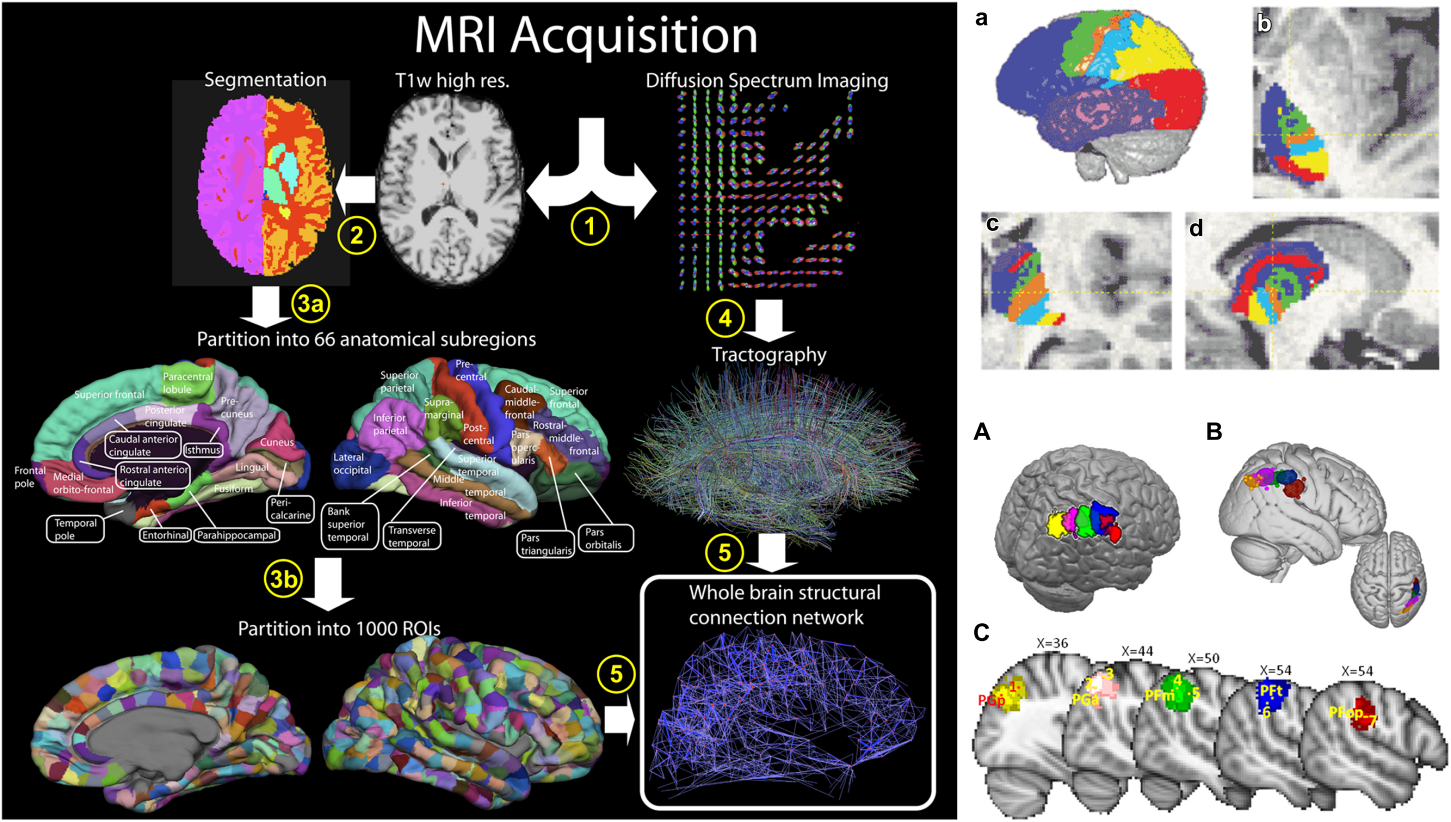
**Two different approaches to the macroscale mapping of the brain anatomical connectivity.** The left column shows the “connectome approach” processing pipeline. In this case, two processing streams are used to define the nodes (cortical areas) and the edges (fiber tracks), obtained using T1-weighted image segmentation and dMRI-based tractography, respectively. The right column shows the “clustering approach.” In this case, the nodes are defined from clustering connectivity information obtained from tractography. Parcellations of the thalamus **(top row)** and of the inferior parietal lobule (IPL, **bottom row**) are shown as examples of anatomical delineation of subcortical and cortical structures, respectively. Right column figure has originally been published in Hagmann et al. (2008). Left column, top row figure is reprinted by permission from Macmillan Publishers Ltd (Behrens et al., 2003a). Left column, bottom row figure is republished with permission of the Society for Neuroscience, from Mars et al. (2011); permission conveyed through Copyright Clearance Center, Inc.

It is therefore possible to map the macrostructural connection organization of the entire brain using dMRI and tractography in two different ways. First, when the origin and termination sites of tracts have been previously defined, tractography can be used to reconstruct the entire macroscopic wiring map of the brain, i.e., the macroscopic connectome, *in vivo* and non-invasively, i.e., the “connectome approach” (**Figure 5**; Hagmann et al., 2008; Van Essen and Ugurbil, 2012; Sotiropoulos et al., 2013). Secondly, the reverse approach is to define cortical areas using clustering approaches based on large scale brain structural connectivity profiles previously obtained using dMRI-based tractography, i.e., the “clustering approach” (**Figure 5**; Behrens et al., 2003a; Tomassini et al., 2007; Perrin et al., 2008; Mars et al., 2011). The clustering approach has led to the identification and subdivision of several cortical (Tomassini et al., 2007; Perrin et al., 2008; Mars et al., 2011) and subcortical (Behrens et al., 2003a) areas. More recently, the clustering of large parts or the entire human neocortex into areas has been attempted using this approach (Perrin et al., 2008; Gorbach et al., 2011).

The connectome matrix can be either weighted or binarized. This means that the edges connecting the different nodes of the connectome can either indicate the strength of a connection between two different cortical areas or simply indicate the existence or lack of a specific neural pathway. The typical approach consists in initially obtaining a weighted connectivity matrix that is then thresholded and binarized. Methods of computing the weights include mean path length between the nodes, averaged FA or MD values along the tracts, or probabilistic and deterministic streamline counts (Li et al., 2012). As discussed in the previous section, estimating ‘connectivity’ between areas using dMRI is not straightforward. As a consequence, the definition of the weights and the choice of the threshold used to binarize the connectivity matrix can dramatically influence the metrics obtained from connectome analysis (Bastiani et al., 2012). Moreover, the matrix representing the connectome can be symmetric or asymmetric. An asymmetric matrix indicates that connection weights are different when tracking from ROI A to ROI B in respect to those obtained when tracking in the opposite direction. Since connectivity indices estimated from dMRI-based tractography do not reflect the direction of the connections between two areas, it is common practice to symmetrize the connectivity matrix, for instance by taking the maximum of the two weights between regions.

In the connectome approach, the application of network analysis to the connectome graph has allowed neuroscientists to describe different properties of cortical areas based on their structural connectivity profiles, such as their degree of segregation based on clustering approaches or their centrality in the communication network (Rubinov and Sporns, 2010). It was recently shown that some cortical regions form a so-called ‘rich club,’ in the sense that those areas are not only very central areas connected to many other areas (i.e., brain hubs), but that these rich club members are also very strongly connected to each other (van den Heuvel and Sporns, 2011). These findings can be used to study several pathological conditions by bringing the analysis to a new level of abstraction (van den Heuvel et al., 2010), in which different brains can be compared based on their connectivity profiles rather than using a common standard template. This can even be used to compare the macroscale connectomes of two different species and identify similarities and differences in their connectivity profiles (Goulas et al., 2014).

Several issues continue to exist in the pursuit of whole brain tractography in particular and caution should be exercised when interpreting connectivity results obtained using diffusion MRI tractography (Thomas et al., 2014). The selection of tractography algorithm in reconstructing the macroscale connectome has a considerable effect on resulting connectivity estimates (Bastiani et al., 2012; Pestilli et al., 2014). Estimating connectivity indices that are both accurate and specific is not straightforward. Tractography algorithms can reconstruct streamlines that are well correlated to large axonal bundles or tracts, but false positives and false negatives will often exist. When using deterministic tractography algorithms, the number of streamlines that successfully reached ROI B when seeded from ROI A is usually taken as a measure of connectivity. However, this result is highly dependent on the resolution of the dMRI data and on the initial parameters set by the user, such as the position of the ROIs, the step size, angular threshold and the intra-voxel diffusion model. Moreover, connection probabilities that are estimated when using probabilistic tractography algorithms are not a measure of true anatomical connectivity. The term ‘probabilistic’ that is used to identify the class of tractography algorithms points at the estimation of uncertainty in fiber orientation obtained with a particular intra-voxel diffusion model. Probabilistic tractography algorithms randomly select the next propagation step within a confidence interval around the estimated orientation, incorporating the effect of noise on the local estimation of the main axonal orientation. The final output of such algorithms will still be a streamline count between two different ROIs, be it a count of probabilistically propagated streamlines. Moreover, the distance between two ROIs influences these total counts. The further apart two ROIs are, the more difficult it will be for streamlines to propagate from one to another (Jones, 2010). Furthermore, fibers can cross or kiss within a voxel, and these configurations can be resolved, though not clearly distinguished, when using HARDI measurements and sophisticated orientation modeling. Fibers can also bend or fan within a voxel, and these configurations will be undistinguishable when looking at the dMRI signal of a single voxel (for a review, see: Jbabdi and Johansen-Berg, 2011). Recent works have tried to solve this issue by fitting helical curves between neighboring voxels to determine whether fibers within them will fan or bend (Savadjiev et al., 2006, 2008). Moreover, it is also possible to determine the polarity of the fanning (i.e., fanning-in or fanning-out), which is very useful when performing tractography (Campbell et al., 2014). Another suggested approach is to model the intravoxel diffusion fODFs while breaking the assumption of symmetry. Since the recorded dMRI signal is symmetric in a three-dimensional space, most diffusion modeling techniques try to estimate a symmetric PDF. Based on the fact that neuronal fibers are continuous between adjacent voxels (i.e., a fiber that leaves a voxel with a certain orientation should enter the next one with the same orientation), a recent work has investigated the benefits of modeling local fODFs as asymmetric functions (Reisert and Kiselev, 2011; Reisert et al., 2012).

## Mesoscopic Brain Organization: Cortical Columns and Layers

Zooming in to the mesoscopic scale the brain again offers very clear and coherent patterns of structural organization. From this viewpoint it is especially interesting to observe the architecture within cerebral neocortex. Roughly speaking, a grid-like structure can be defined. With respect to the pial surface, neurons in gray matter are organized radially (along the depth of the two to four millimeter thick cortex) in columns and tangentially (along the two dimension surface of the cortex) in layers. A single cortical column spans the cortical layers vertically, from the pial surface to the border between white and gray matter and, when combined with other columns, forms a so-called macrocolumn (Mountcastle, 1997). The definition of functional columns arose from electrophysiological studies reporting that, when moving electrodes perpendicularly to the gray matter surface, there was a very strong degree of consistency in the receptive field of neurons in the primary visual cortex (Hubel and Wiesel, 1962, 1968; Mountcastle, 1997). The anatomical characterization of neocortical columns is notoriously difficult (Rakic, 2008), except perhaps in rare cases such as the barrel fields in rodent somatosensory cortex, leading to doubts about the anatomical significance of the cortical column concept (Horton and Adams, 2005; da Costa and Martin, 2010). The laminar organization of the cortex is much more neuroanatomically defined. From the cytoarchitectural point of view, the cortex is organized in layers, which are tangential to the pial surface and are characterized by different densities, sizes and morphology of cell bodies.

Since both cyto- and myeloarchitectonics show a layered organization of the cortical mantle, the delineation of cortical areas can be inferred from different patterns of cell bodies as well as from changes in intracortical fiber configurations. However, the relationship between cytoarchitecture and myeloarchitecture in the cortex is still not clear. Hellwig (1993) showed, using simulations, that it is possible to predict the myelin content over the depth (i.e., layers) of the cortex from cytoarchitecture using two assumptions, namely that neurons with a bigger cell body size contribute more to the intracortical myelin content and that the distribution of axon collaterals can be quantified using a simple model. This model relates the amount of axon collaterals originating from pyramidal neurons with their distance from the respective cell body.

Each cortical layer tends to be characterized by different afferent and efferent projections. Moreover, the brain can be seen as a hierarchically organized system, where the hierarchical level of a cortical area is determined by the distance (in number of synaptic connections) from sensory areas. There are three main patterns of laminar projections within the hierarchically organized cortical system, namely ascending, lateral, and descending projections (Felleman and Van Essen, 1991). These pathways tend to have relatively specific laminar origin and termination patterns. Ascending pathways coming from hierarchically lower cortical areas tend to terminate in layer 4, lateral projections coming from hierarchically similar cortical areas terminate across all layers in a columnar fashion and descending projections coming from hierarchically higher cortical areas avoid layer 4 and terminate both in superficial and deep layers. Generally, infragranular layers (layers V and VI) are those that mainly project to sub-cortical areas, while supragranular layers (layers II and III) are connected to other cortical locations (Jones, 1981; Fuster, 2005).

**FIGURE 6:**
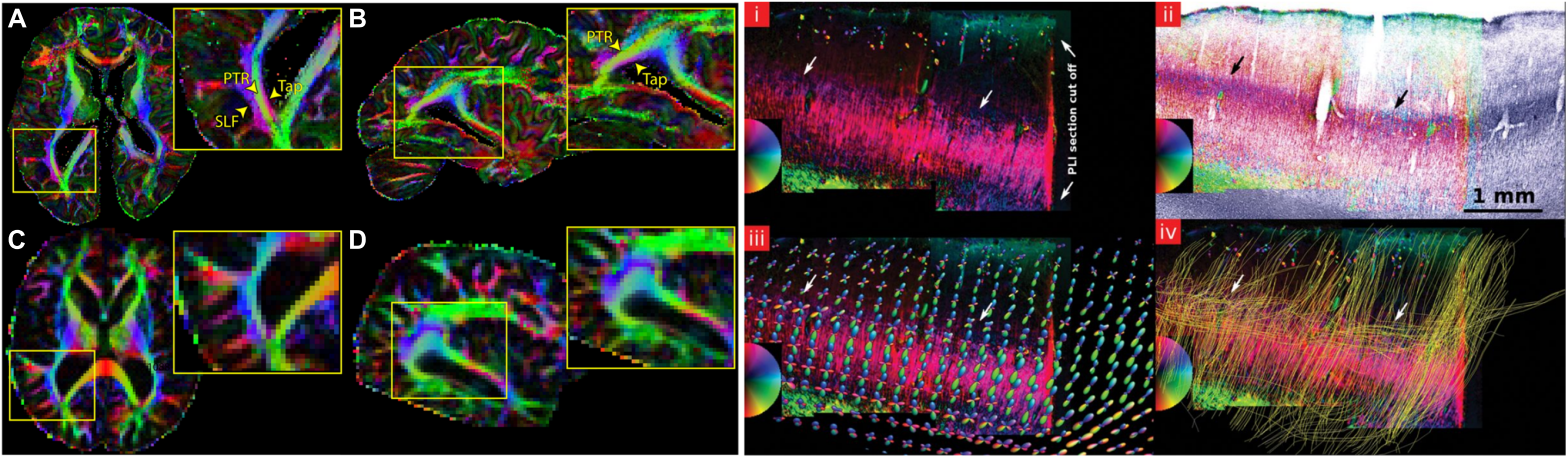
**Examples showing that dMRI can be a valuable tool to investigate the mesoscopic organization of the brain.** Recent advancements in sequence programming and MRI hardware have allowed the acquisition of whole-brain post-mortem samples at sub-millimeter resolution. The left panel shows a comparison between a whole-brain post-mortem volume acquired at 0.73 mm isotropic resolution **(A,B)** versus a typical *in vivo* acquisition at 2 mm isotopic resolution **(C,D)**. The insets show that the gain in resolution allows a better segregation of fiber tracts that are usually not easily distinguishable *in vivo* acquisitions, such as the tapetum of the corpus callosum (Tap), the posterior thalamic radiation (PTR) and the superior longitudinal fasciculus (SLF). The right panel shows an application of dMRI-based tractography to the study of intracortical connectivity in primary visual cortex. (i) shows a PLI section containing the stria of Gennari, (ii) is the same slice overlaid on a myelin stained section of the same tissue, (iii) compares the color-coded PLI section in (i) with the estimated fODFs obtained using CSD and (iv) the results of tractography ran after estimating the fODFs, where the cortically tangential component of the stria of Gennari is correctly reconstructed. The figure on the left has originally been published in Miller et al. (2011). The figure on the right is from Leuze et al. (2014), reprinted by permission of Oxford University Press.

Diffusion MRI can be used to map the mesoscopic organization of the brain (McKinstry et al., 2002). The high resolution needed to look at the mesoscopic organization of the brain can be achieved by acquiring data from excised tissue samples (D’Arceuil et al., 2007). Although long scanning times can be achieved in *ex vivo* acquisitions to increase SNR, different temperature conditions and tissue fixation effects have an influence on dMRI contrast. This is usually reflected in lower FA and lower MD values, as well as a greater challenges in the tracking of axonal fibers (D’Arceuil and de Crespigny, 2007). Post-mortem dMRI imaging has recently progressed to studying the structural organization of the entire human brain at a voxel resolution of 0.7 mm isotropic (**Figure 6**; Miller et al., 2011). Other studies have focused on smaller tissue samples to reduce the necessary field of view and increase isotropic voxel size, bringing it to 0.2–0.4 mm. Leuze et al. (2014) describe the intra and inter-laminar connectivity within post-mortem tissue samples of primary visual cortex using CSD-based tractography (**Figure 6**). Furthermore, the organization of primary visual cortex, where the highly myelinated stria of Gennari can be found, was investigated using post-mortem dMRI (Kleinnijenhuis et al., 2013). All the aforementioned results show how powerful dMRI-based techniques are to resolve complex anatomical features in the human brain. Moreover, in studies of post-mortem tissue samples, dMRI volumes can be combined with histological investigations of the same tissue. This is potentially very useful especially to validate the results of tractography and to better tune the available algorithms (Seehaus et al., 2013).

## Microscopic Brain Organization: Axonal Densities and Diameters

From the microscopic viewpoint, both white and gray matter show a wide range of different patterns in their structural organization. Within white matter, axons themselves are characterized by different diameters and packing densities. In the corpus callosum of both macaque (Lamantia and Rakic, 1990) and human (Aboitiz et al., 1992), several clusters of tracts have been identified. Using light microscopy, Aboitiz et al. (1992) examined the corpus callosum of several individuals and found that thin axons are denser in the genu. Furthermore, their density decreases moving toward the posterior midbody and increases again in the splenium. On the other hand, thicker fibers exhibit the opposite pattern, with a maximum density in the posterior midbody of the corpus callosum. Interestingly, the authors were not able to find any correlation between callosal volume and fiber density and between gender and density.

Diffusion MRI has recently proven to be successful in mapping brain microstructural architecture. Several new biophysical markers have been estimated that show strong correlation to information such as axonal diameters, axonal density and fiber dispersion both *in vivo* and post-mortem. These advances have been achieved by introducing new modeling approaches that aim to estimate and separate the contributions of different biological compartments within the white matter, most importantly the restricted intra- and hindered extra-axonal compartments (Assaf and Basser, 2005; Panagiotaki et al., 2012; Zhang et al., 2012). If modeled correctly, these two separate water pools are characterized by different signal profiles in standard pulsed gradient spin echo (PGSE) experiments (Stanisz et al., 1997). This difference in signal properties can be used to fit dMRI microstructural compartment models of white matter to the acquired data. Depending on the type of acquisition (e.g., how many *b*-values) and selected model, it is then possible to infer the microstructural characteristics of the tissue from model parameters such as compartment volume fractions (for a review, see: Panagiotaki et al., 2012). This makes it possible to infer patterns of axonal densities in the human brain as well as relative axonal diameter distributions, both *in vivo* and *ex vivo* (Assaf et al., 2008; Barazany et al., 2009; Alexander et al., 2010; Zhang et al., 2012).

To study conduction velocities and signal transmission in the nervous system, anatomists have been looking to the so-called g-ratio, which is the ratio between the membrane-to-membrane axonal diameter and the myelinated fiber diameter. Rushton (1951) was the first to describe a mathematical approach to define the optimal g-ratio in the nervous system, which predicted to be 0.6. His theoretical framework was solely based on the velocity of signal transmission, and recent studies have both challenged and improved this framework in the central nervous system of the rat (Chomiak and Hu, 2009). The g-ratio represents an important marker for detecting and studying progression of diseases, axonal plasticity, and development. As recently shown by Zikopoulos and Barbas (2011), the comparison of different microstructural indices such as the *g*-ratio and axonal diameters between individual axons of the frontal cortex of both human and non-human primates can lead to the identification of several differences and similarities between different species. Such findings are fundamental to follow and explain the evolutionary paths followed by different species and represent a necessary step to understand the neuroanatomical underpinning of neurological diseases. The dMRI models discussed above have also allowed estimating the axonal g-ratio *in vivo*. By looking at the ratio between the intra-axonal volume compartment, estimated using the neurite orientation dispersion and density imaging technique (NODDI; Zhang et al., 2012) and myelin water fraction, Stikov et al. (2014) have shown that it the g-ratio can be computed *in vivo* from human dMRI data.

## Conclusion and Future Directions

Technical advancements in the world of microscopy are constantly increasing both in level of detail and the field of view that can be investigated. Recent efforts have already shown the potential of classical cytoarchitectonic staining of an entire brain at the very high resolution of 20 microns (Amunts et al., 2013) with more individual datasets currently underway. Combining such maps with observer-independent techniques to demarcate architecturally different cortical areas (Schleicher et al., 1999) will allow identifying new common structural principles which are shared between individuals and which could not be described in earlier works based on single subjects (Brodmann, 1909; Talairach and Tournoux, 1988). Furthermore, it has been shown that three-dimensional structural connections can be investigated in larger tissue samples after having made the tissue transparent with optical clearing techniques (Chung and Deisseroth, 2013).

Validation of dMRI-based techniques is still an important issue. The combination of whole brain post-mortem findings obtained from techniques such as PLI (Axer et al., 2011b) with tractography results might represent a very good way to address this. Both techniques in principle allow for a whole brain three-dimensional reconstruction of fiber tracts. Here it should be noted that such techniques, although at different intrinsic resolutions, have the same basic tractography problem to solve, starting from local fiber directions. Another technique which can help in validating dMRI-based tractography results is optical coherence tomography (OCT; Huang et al., 1991). As an advantage over PLI, OCT does not require the tissue to be sectioned and it is therefore less prone to deformation artifacts. OCT provides both cyto (Magnain et al., 2014) and myeloarchitectural information at resolutions of tens of microns. This three-dimensional volumetric information can provide a gold standard when coregistered to dMRI volumes (Wang et al., 2011). At the microstructural mapping side, there is also the need to improve the sensitivity and the specificity of estimated microstructural indices and reconstructed axonal pathways (Bells et al., 2011).

Finally, consortium projects including large population studies have recently started mapping structural and functional macroscale connectomes of adult human brains (Van Essen and Ugurbil, 2012; Sotiropoulos et al., 2013) and newborns (http://www.developingconnectome.org/) at the population level and at very high resolution. Making these data available to the whole scientific community must become a fundamental prior to any future study aimed at mapping the structural architecture of the brain.

## Conflict of Interest Statement

The authors declare that the research was conducted in the absence of any commercial or financial relationships that could be construed as a potential conflict of interest.

## References

[B1] Aboitiz F. Scheibel A. B. Fisher R. S. Zaidel E. (1992). Fiber composition of the human corpus callosum. *Brain Res.* 598 143–153. 10.1016/0006-8993(92)90178-C 1486477

[B2] Alexander D. C. Barker G. J. Arridge S. R. (2002). Detection and modeling of non-Gaussian apparent diffusion coefficient profiles in human brain data. *Magn. Reson. Med.* 48 331–340. 10.1002/mrm.10209 12210942

[B3] Alexander D. C. Hubbard P. L. Hall M. G. Moore E. A. Ptito M. Parker G. J. (2010). Orientationally invariant indices of axon diameter and density from diffusion MRI. *Neuroimage* 52 1374–1389. 10.1016/j.neuroimage.2010.05.043 20580932

[B4] Amunts K. Lenzen M. Friederici A. D. Schleicher A. Morosan P. Palomero-Gallagher N. (2010). Broca’s region: novel organizational principles and multiple receptor mapping. *PLoS Biol.* 8:e1000489 10.1371/journal.pbio.1000489 PMC2943440 20877713

[B5] Amunts K. Lepage C. Borgeat L. Mohlberg H. Dickscheid T. Rousseau M. E. (2013). BigBrain: an ultrahigh-resolution 3D human brain model. *Science* 340 1472–1475. 10.1126/science.1235381 23788795

[B6] Assaf Y. Basser P. J. (2005). Composite hindered and restricted model of diffusion (CHARMED) MR imaging of the human brain. *Neuroimage* 27 48–58. 10.1016/j.neuroimage.2005.03.042 15979342

[B7] Assaf Y. Blumenfeld-Katzir T. Yovel Y. Basser P. J. (2008). AxCaliber: a method for measuring axon diameter distribution from diffusion MRI. *Magn. Reson. Med.* 59 1347–1354. 10.1002/mrm.21577 18506799 PMC4667732

[B8] Axer H. Axer M. Krings T. Keyserlingk D. G. (2001). Quantitative estimation of 3-D fiber course in gross histological sections of the human brain using polarized light. *J. Neurosci. Methods* 105 121–131. 10.1016/S0165-0270(00)00349-6 11275269

[B9] Axer H. Beck S. Schuchardt F. Heepe J. Flücken A. Axer M. (2011a). Microstructural analysis of human white matter architecture using polarized light imaging: views from neuroanatomy. *Front. Neuroinform.* 5:28 10.3389/fninf.2011.00028 PMC3215979 22110430

[B10] Axer M. Grassel D. Kleiner M. Dammers J. Dickscheid T. Reckfort J. (2011b). High-resolution fiber tract reconstruction in the human brain by means of three-dimensional polarized light imaging. *Front. Neuroinform.* 5:34 10.3389/fninf.2011.00034 PMC3248698 22232597

[B11] Axer H. Keyserlingk D. G. (2000). Mapping of fiber orientation in human internal capsule by means of polarized light and confocal scanning laser microscopy. *J. Neurosci. Methods* 94 165–175. 10.1016/S0165-0270(99)00132-6 10661836

[B12] Bakker R. Wachtler T. Diesmann M. (2012). CoCoMac 2.0 and the future of tract-tracing databases. *Front. Neuroinform.* 6:30 10.3389/fninf.2012.00030 PMC3530798 23293600

[B13] Barazany D. Basser P. J. Assaf Y. (2009). In vivo measurement of axon diameter distribution in the corpus callosum of rat brain. *Brain* 132 1210–1220. 10.1093/brain/awp042 19403788 PMC2677796

[B14] Basser P. J. Mattiello J. Lebihan D. (1994). MR diffusion tensor spectroscopy and imaging. *Biophys. J.* 66 259–267. 10.1016/S0006-3495(94)80775-1 8130344 PMC1275686

[B15] Basser P. J. Pajevic S. Pierpaoli C. Duda J. Aldroubi A. (2000). In vivo fiber tractography using DT-MRI data. *Magn. Reson. Med*. 44 625–632. 10.1002/1522-2594(200010)44:4<625::AID-MRM17>3.0.CO;2-O 11025519

[B16] Basser P. J. Pierpaoli C. (1996). Microstructural and physiological features of tissues elucidated by quantitative-diffusion-tensor MRI. *J. Magn. Reson. B* 111 209–219. 10.1006/jmrb.1996.0086 8661285

[B17] Bastiani M. Shah N. J. Goebel R. Roebroeck A. (2012). Human cortical connectome reconstruction from diffusion weighted MRI: the effect of tractography algorithm. *Neuroimage* 62 1732–1749. 10.1016/j.neuroimage.2012.06.002 22699045

[B18] Behrens T. E. Berg H. J. Jbabdi S. Rushworth M. F. Woolrich M. W. (2007). Probabilistic diffusion tractography with multiple fibre orientations: what can we gain? *Neuroimage* 34 144–155. 10.1016/j.neuroimage.2006.09.018 17070705 PMC7116582

[B19] Behrens T. E. Johansen-Berg H. Woolrich M. W. Smith S. M. Wheeler-Kingshott C. A. Boulby P. A. (2003a). Non-invasive mapping of connections between human thalamus and cortex using diffusion imaging. *Nat. Neurosci.* 6 750–757. 10.1038/nn1075 12808459

[B20] Behrens T. E. Woolrich M. W. Jenkinson M. Johansen-Berg H. Nunes R. G. Clare S. (2003b). Characterization and propagation of uncertainty in diffusion-weighted MR imaging. *Magn. Reson. Med.* 50 1077–1088. 10.1002/mrm.10609 14587019

[B21] Bells S. Cercignani M. Deoni S. Assaf Y. Pasternak O. Evans C. J. (2011). “Tractometry: comprehensive multi-modal quantitative assessment of white matter along specific tracts,” in *Proceedings of the 19th Annual Meeting of International Society for Magnetic Resonance in Medicine* Montreal, QC.

[B22] Bezgin G. Vakorin V. A. Van Opstal A. J. Mcintosh A. R. Bakker R. (2012). Hundreds of brain maps in one atlas: registering coordinate-independent primate neuro-anatomical data to a standard brain. *Neuroimage* 62 67–76. 10.1016/j.neuroimage.2012.04.013 22521477

[B23] Bonin G. V. Bailey P. (1947). *The Neocortex of Macaca Mulatta*. Urbana: University of Illinois Press.

[B24] Bressler S. L. Menon V. (2010). Large-scale brain networks in cognition: emerging methods and principles. *Trends Cogn. Sci.* 14 277–290. 10.1016/j.tics.2010.04.004 20493761

[B25] Brodmann K. (1909). *Vergleichende Lokalisationslehre der Grosshirnrinde in ihren Prinzipien dargestellt auf Grund des Zellenbaues*. Leipzig: Johann Ambrosius Barth.

[B26] Callaghan P. T. Eccles C. D. Xia Y. (1988). NMR microscopy of dynamic displacements: k-space and q-space imaging. *J. Phys. E Sci. Instrum.* 21 820 10.1088/0022-3735/21/8/017

[B27] Campbell J. S. Momayyezsiahkal P. Savadjiev P. Leppert I. R. Siddiqi K. Pike G. B. (2014). Beyond crossing fibers: bootstrap probabilistic tractography using complex subvoxel fiber geometries. *Front. Neurol.* 5:216 10.3389/fneur.2014.00216 PMC4211389 25389414

[B28] Caspers S. Axer M. Caspers J. Jockwitz C. Jütten K. Reckfort J. (2015). Target sites for transcallosal fibers in human visual cortex – a combined diffusion and polarized light imaging study. *Cortex* 10.1016/j.cortex.2015.01.009 [Epub ahead of print]. 25697048

[B29] Catani M. Dell’acqua F. Vergani F. Malik F. Hodge H. Roy P. (2012). Short frontal lobe connections of the human brain. *Cortex* 48 273–291. 10.1016/j.cortex.2011.12.001 22209688

[B30] Catani M. Howard R. J. Pajevic S. Jones D. K. (2002). Virtual in vivo interactive dissection of white matter fasciculi in the human brain. *Neuroimage* 17 77–94. 10.1006/nimg.2002.1136 12482069

[B31] Catani M. Thiebaut de Schotten M. (2008). A diffusion tensor imaging tractography atlas for virtual in vivo dissections. *Cortex* 44 1105–1132. 10.1016/j.cortex.2008.05.004 18619589

[B32] Catani M. Thiebaut De Schotten M. Slater D. Dell’acqua F. (2013). Connectomic approaches before the connectome. *Neuroimage* 80 2–13. 10.1016/j.neuroimage.2013.05.109 23735262

[B33] Chomiak T. Hu B. (2009). What is the optimal value of the g-Ratio for myelinated fibers in the rat CNS? A theoretical approach. *PLoS ONE* 4:e7754 10.1371/journal.pone.0007754 PMC2771903 19915661

[B34] Chung K. Deisseroth K. (2013). CLARITY for mapping the nervous system. *Nat. Methods* 10 508–513. 10.1038/nmeth.2481 23722210

[B35] Conturo T. E. Lori N. F. Cull T. S. Akbudak E. Snyder A. Z. Shimony J. S. (1999). Tracking neuronal fiber pathways in the living human brain. *Proc. Natl. Acad. Sci. U.S.A.* 96 10422–10427. 10.1073/pnas.96.18.10422 10468624 PMC17904

[B36] Craddock R. C. Jbabdi S. Yan C. G. Vogelstein J. T. Castellanos F. X. Di Martino A. (2013). Imaging human connectomes at the macroscale. *Nat. Methods* 10 524–539. 10.1038/nmeth.2482 23722212 PMC4096321

[B37] D’Arceuil H. de Crespigny A. (2007). The effects of brain tissue decomposition on diffusion tensor imaging and tractography. *Neuroimage* 36 64–68. 10.1016/j.neuroimage.2007.02.039 17433879 PMC4039353

[B38] D’Arceuil H. E. Westmoreland S. De Crespigny A. J. (2007). An approach to high resolution diffusion tensor imaging in fixed primate brain. *Neuroimage* 35 553–565. 10.1016/j.neuroimage.2006.12.028 17292630

[B39] da Costa N. M. Martin K. A. (2010). Whose cortical column would that be? *Front. Neuroanat.* 4:16 10.3389/fnana.2010.00016 PMC2904586 20640245

[B40] Dammers J. Axer M. Grassel D. Palm C. Zilles K. Amunts K. (2010). Signal enhancement in polarized light imaging by means of independent component analysis. *Neuroimage* 49 1241–1248. 10.1016/j.neuroimage.2009.08.059 19733674

[B41] Dammers J. Breuer L. Axer M. Kleiner M. Eiben B. Grassel D. (2012). Automatic identification of gray and white matter components in polarized light imaging. *Neuroimage* 59 1338–1347. 10.1016/j.neuroimage.2011.08.030 21875673

[B42] Dong Q. Welsh R. C. Chenevert T. L. Carlos R. C. Maly-Sundgren P. Gomez-Hassan D. M. (2004). Clinical applications of diffusion tensor imaging. *J. Magn. Reson. Imaging* 19 6–18. 10.1002/jmri.10424 14696215

[B43] Eickhoff S. B. Rottschy C. Kujovic M. Palomero-Gallagher N. Zilles K. (2008). Organizational principles of human visual cortex revealed by receptor mapping. *Cereb. Cortex* 18 2637–2645. 10.1093/cercor/bhn024 18321873 PMC2733321

[B44] Einstein A. (1905). Über die von der molekularkinetischen theorie der wärme geforderte bewegung von in ruhenden flüssigkeiten suspendierten teilchen. *Ann. Phys.* 322 549–560. 10.1002/andp.19053220806

[B45] Felleman D. J. Van Essen D. C. (1991). Distributed hierarchical processing in the primate cerebral cortex. *Cereb. Cortex* 1 1–47. 10.1093/cercor/1.1.1 1822724

[B46] Frank L. R. (2002). Characterization of anisotropy in high angular resolution diffusion-weighted MRI. *Magn. Reson. Med.* 47 1083–1099. 10.1002/mrm.10156 12111955

[B47] Fuster J. M. (2005). “Neurobiology of cortical networks,” in *Cortex and Mind* (New York, NY: Oxford University Press). 10.1093/acprof:oso/9780195300840.001.0001

[B48] Geyer S. Matelli M. Luppino G. Schleicher A. Jansen Y. Palomero-Gallagher N. (1998). Receptor autoradiographic mapping of the mesial motor and premotor cortex of the macaque monkey. *J. Comp. Neurol.* 397 231–250. 10.1002/(SICI)1096-9861(19980727)397:2<231::AID-CNE6>3.0.CO;2-1 9658286

[B49] Gorbach N. S. Schutte C. Melzer C. Goldau M. Sujazow O. Jitsev J. (2011). Hierarchical information-based clustering for connectivity-based cortex parcellation. *Front. Neuroinform.* 5:18 10.3389/fninf.2011.00018 PMC3178812 21977015

[B50] Goulas A. Bastiani M. Bezgin G. Uylings H. B. Roebroeck A. Stiers P. (2014). Comparative analysis of the macroscale structural connectivity in the macaque and human brain. *PLoS Comput. Biol.* 10:e1003529 10.1371/journal.pcbi.1003529 PMC3967942 24676052

[B51] Hagmann P. Cammoun L. Gigandet X. Meuli R. Honey C. J. Wedeen V. J. (2008). Mapping the structural core of human cerebral cortex. *PLoS Biol.* 6:e159 10.1371/journal.pbio.0060159 PMC2443193 18597554

[B52] Hagmann P. Jonasson L. Maeder P. Thiran J. P. Wedeen V. J. Meuli R. (2006). Understanding diffusion MR imaging techniques: from scalar diffusion-weighted imaging to diffusion tensor imaging and beyond. *Radiographics* 26(Suppl. 1) S205–S223. 10.1148/rg.26si065510 17050517

[B53] Hellwig B. (1993). How the myelin picture of the human cerebral cortex can be computed from cytoarchitectural data. a bridge between von economo and vogt. *J. Hirnforsch.* 34 387–402. 8270790

[B54] Horton J. C. Adams D. L. (2005). The cortical column: a structure without a function. *Philos. Trans. R. Soc. Lond. B Biol. Sci.* 360 837–862. 10.1098/rstb.2005.1623 15937015 PMC1569491

[B55] Huang D. Swanson E. A. Lin C. P. Schuman J. S. Stinson W. G. Chang W. (1991). Optical coherence tomography. *Science* 254 1178–1181. 10.1126/science.1957169 1957169 PMC4638169

[B56] Hubel D. H. Wiesel T. N. (1962). Receptive fields, binocular interaction and functional architecture in the cat’s visual cortex. *J. Physiol.* 160 106–154. 10.1113/jphysiol.1962.sp006837 14449617 PMC1359523

[B57] Hubel D. H. Wiesel T. N. (1968). Receptive fields and functional architecture of monkey striate cortex. *J. Physiol.* 195 215–243. 10.1113/jphysiol.1968.sp008455 4966457 PMC1557912

[B58] Iturria-Medina Y. Canales-Rodriguez E. J. Melie-Garcia L. Valdes-Hernandez P. A. Martinez-Montes E. Aleman-Gomez Y. (2007). Characterizing brain anatomical connections using diffusion weighted MRI and graph theory. *Neuroimage* 36 645–660. 10.1016/j.neuroimage.2007.02.012 17466539

[B59] Jansons K. M. Alexander D. C. (2003). Persistent Angular Structure: new insights from diffusion MRI data. Dummy version. *Inf. Process. Med. Imaging* 18 672–683. 10.1007/978-3-540-45087-0_56 15344497

[B60] Jbabdi S. Johansen-Berg H. (2011). Tractography: where do we go from here? *Brain Connect*. 1 169–183. 10.1089/brain.2011.0033 22433046 PMC3677805

[B61] Jbabdi S. Woolrich M. W. Andersson J. L. Behrens T. E. (2007). A Bayesian framework for global tractography. *Neuroimage* 37 116–129. 10.1016/j.neuroimage.2007.04.039 17543543

[B62] Jeurissen B. Leemans A. Tournier J. D. Jones D. K. Sijbers J. (2013). Investigating the prevalence of complex fiber configurations in white matter tissue with diffusion magnetic resonance imaging. *Hum. Brain Mapp.* 34 2747–2766. 10.1002/hbm.22099 22611035 PMC6870534

[B63] Jones D. K. (2003). Determining and visualizing uncertainty in estimates of fiber orientation from diffusion tensor MRI. *Magn. Reson. Med.* 49 7–12. 10.1002/mrm.10331 12509814

[B64] Jones D. K. (2010). Challenges and limitations of quantifying brain connectivity in vivo with diffusion MRI. *Imaging Med.* 2 341–355. 10.2217/iim.10.21

[B65] Jones E. G. (1981). “Anatomy of cerebral cortex: columnar input-output organization,” in *The Organization of the Cerebral Cortex* eds Schmitt F. O. Worden F. G. Adelman G. Dennis S. G. (Cambridge, MA: MIT Press).

[B66] Khayal I. S. Vandenberg S. R. Smith K. J. Cloyd C. P. Chang S. M. Cha S. (2011). MRI apparent diffusion coefficient reflects histopathologic subtype, axonal disruption, and tumor fraction in diffuse-type grade II gliomas. *Neuro Oncol.* 13 1192–1201. 10.1093/neuonc/nor122 21865401 PMC3199150

[B67] Kleiner M. Axer M. Grassell D. Reckfort J. Pietrzyk U. Amunts K. (2012). Classification of ambiguous nerve fiber orientations in 3D polarized light imaging. *Med. Image Comput. Comput. Assist. Interv.* 15 206–213. 10.1007/978-3-642-33415-3_26 23285553

[B68] Kleinnijenhuis M. Zerbi V. Kusters B. Slump C. H. Barth M. Van Cappellen Van Walsum A. M. (2013). Layer-specific diffusion weighted imaging in human primary visual cortex in vitro. *Cortex* 49 2569–2582. 10.1016/j.cortex.2012.11.015 23347559

[B69] Kotter R. Wanke E. (2005). Mapping brains without coordinates. *Philos. Trans. R. Soc. B Biol. Sci.* 360 751–766. 10.1098/rstb.2005.1625 PMC1569487 15971361

[B70] Kreher B. W. Mader I. Kiselev V. G. (2008). Gibbs tracking: a novel approach for the reconstruction of neuronal pathways. *Magn. Reson. Med.* 60 953–963. 10.1002/mrm.21749 18816816

[B71] Lamantia A. S. Rakic P. (1990). Cytological and quantitative characteristics of four cerebral commissures in the rhesus monkey. *J. Comp. Neurol.* 291 520–537. 10.1002/cne.902910404 2329189

[B72] Larsen L. Griffin L. D. Grassel D. Witte O. W. Axer H. (2007). Polarized light imaging of white matter architecture. *Microsc. Res. Tech.* 70 851–863. 10.1002/jemt.20488 17661367

[B73] Leuze C. W. Anwander A. Bazin P. L. Dhital B. Stuber C. Reimann K. (2014). Layer-specific intracortical connectivity revealed with diffusion MRI. *Cereb. Cortex* 24 328–339. 10.1093/cercor/bhs311 23099298 PMC3888365

[B74] Li L. Rilling J. K. Preuss T. M. Glasser M. F. Hu X. (2012). The effects of connection reconstruction method on the interregional connectivity of brain networks via diffusion tractography. *Hum. Brain Mapp.* 33 1894–1913. 10.1002/hbm.21332 21928316 PMC6414228

[B75] Magnain C. Augustinack J. C. Reuter M. Wachinger C. Frosch M. P. Ragan T. (2014). Blockface histology with optical coherence tomography: a comparison with Nissl staining. *Neuroimage* 84 524–533. 10.1016/j.neuroimage.2013.08.072 24041872 PMC4315235

[B76] Markov N. T. Kennedy H. (2013). The importance of being hierarchical. *Curr. Opin. Neurobiol.* 23 187–194. 10.1016/j.conb.2012.12.008 23339864

[B77] Markov N. T. Misery P. Falchier A. Lamy C. Vezoli J. Quilodran R. (2011). Weight consistency specifies regularities of macaque cortical networks. *Cereb. Cortex* 21 1254–1272. 10.1093/cercor/bhq201 21045004 PMC3097985

[B78] Mars R. B. Jbabdi S. Sallet J. O’Reilly J. X. Croxson P. L. Olivier E. (2011). Diffusion-weighted imaging tractography-based parcellation of the human parietal cortex and comparison with human and macaque resting-state functional connectivity. *J. Neurosci.* 31 4087–4100. 10.1523/JNEUROSCI.5102-10.2011 21411650 PMC3091022

[B79] McKinstry R. C. Mathur A. Miller J. H. Ozcan A. Snyder A. Z. Schefft G. L. (2002). Radial organization of developing preterm human cerebral cortex revealed by non-invasive water diffusion anisotropy MRI. *Cereb. Cortex* 12 1237–1243. 10.1093/cercor/12.12.1237 12427675

[B80] Miller K. L. Stagg C. J. Douaud G. Jbabdi S. Smith S. M. Behrens T. E. (2011). Diffusion imaging of whole, post-mortem human brains on a clinical MRI scanner. *Neuroimage* 57 167–181. 10.1016/j.neuroimage.2011.03.070 21473920 PMC3115068

[B81] Morell P. (1984). *Myelin*. New York: Plenum Press 10.1007/978-1-4757-1830-0

[B82] Mori S. Crain B. J. Chacko V. P. Van Zijl P. C. (1999). Three-dimensional tracking of axonal projections in the brain by magnetic resonance imaging. *Ann. Neurol.* 45 265–269. 10.1002/1531-8249(199902)45:2<265::AID-ANA21>3.0.CO;2-3 9989633

[B83] Mori S. van Zijl P. C. (2002). Fiber tracking: principles and strategies – a technical review. *NMR Biomed.* 15 468–480. 10.1002/nbm.781 12489096

[B84] Mountcastle V. B. (1997). The columnar organization of the neocortex. *Brain* 120 (Pt 4) 701–722. 10.1093/brain/120.4.701 9153131

[B85] Nieuwenhuys R. (2013). The myeloarchitectonic studies on the human cerebral cortex of the Vogt-Vogt school, and their significance for the interpretation of functional neuroimaging data. *Brain Struct. Funct.* 218 303–352. 10.1007/s00429-012-0460-z 23076375

[B86] Ozarslan E. Shepherd T. M. Vemuri B. C. Blackband S. J. Mareci T. H. (2006). Resolution of complex tissue microarchitecture using the diffusion orientation transform (DOT). *Neuroimage* 31 1086–1103. 10.1016/j.neuroimage.2006.01.024 16546404

[B87] Panagiotaki E. Schneider T. Siow B. Hall M. G. Lythgoe M. F. Alexander D. C. (2012). Compartment models of the diffusion MR signal in brain white matter: a taxonomy and comparison. *Neuroimage* 59 2241–2254. 10.1016/j.neuroimage.2011.09.081 22001791

[B88] Parker G. J. Alexander D. C. (2005). Probabilistic anatomical connectivity derived from the microscopic persistent angular structure of cerebral tissue. *Philos. Trans. R. Soc. Lond. B Biol. Sci.* 360 893–902. 10.1098/rstb.2005.1639 16087434 PMC1854923

[B89] Parker G. J. Haroon H. A. Wheeler-Kingshott C. A. (2003). A framework for a streamline-based probabilistic index of connectivity (PICo) using a structural interpretation of MRI diffusion measurements. *J. Magn. Reson. Imaging* 18 242–254. 10.1002/jmri.10350 12884338

[B90] Patterson D. M. Padhani A. R. Collins D. J. (2008). Technology insight: water diffusion MRI–a potential new biomarker of response to cancer therapy. *Nat. Clin. Pract. Oncol.* 5 220–233. 10.1038/ncponc1073 18301415

[B91] Perrin M. Cointepas Y. Cachia A. Poupon C. Thirion B. Riviere D. (2008). Connectivity-based parcellation of the cortical mantle using q-ball diffusion imaging. *Int. J. Biomed. Imaging* 2008 368406 10.1155/2008/368406 PMC2288697 18401457

[B92] Pestilli F. Yeatman J. D. Rokem A. Kay K. N. Wandell B. A. (2014). Evaluation and statistical inference for human connectomes. *Nat. Methods* 11 1058–1063. 10.1038/nmeth.3098 25194848 PMC4180802

[B93] Rakic P. (2008). Confusing cortical columns. *Proc. Natl. Acad. Sci. U.S.A.* 105 12099–12100. 10.1073/pnas.0807271105 18715998 PMC2527871

[B94] Reisert M. Kellner E. Kiselev V. G. (2012). About the geometry of asymmetric fiber orientation distributions. *IEEE Trans. Med. Imaging* 31 1240–1249. 10.1109/TMI.2012.2187916 22345527

[B95] Reisert M. Kiselev V. G. (2011). Fiber continuity: an anisotropic prior for ODF estimation. *IEEE Trans. Med. Imaging* 30 1274–1283. 10.1109/TMI.2011.2112769 21317082

[B96] Reisert M. Mader I. Anastasopoulos C. Weigel M. Schnell S. Kiselev V. (2011). Global fiber reconstruction becomes practical. *Neuroimage* 54 955–962. 10.1016/j.neuroimage.2010.09.016 20854913

[B97] Rubinov M. Sporns O. (2010). Complex network measures of brain connectivity: uses and interpretations. *Neuroimage* 52 1059–1069. 10.1016/j.neuroimage.2009.10.003 19819337

[B98] Rushton W. A. (1951). A theory of the effects of fibre size in medullated nerve. *J. Physiol.* 115 101–122. 10.1113/jphysiol.1951.sp004655 14889433 PMC1392008

[B99] Sanides F. (1962). [Architectonics of the human frontal lobe of the brain. With a demonstration of the principles of its formation as a reflection of phylogenetic differentiation of the cerebral cortex]. *Monogr. Gesamtgeb. Neurol. Psychiatr.* 98 1–201. 13976313

[B100] Savadjiev P. Campbell J. S. Descoteaux M. Deriche R. Pike G. B. Siddiqi K. (2008). Labeling of ambiguous subvoxel fibre bundle configurations in high angular resolution diffusion MRI. *Neuroimage* 41 58–68. 10.1016/j.neuroimage.2008.01.028 18367409

[B101] Savadjiev P. Campbell J. S. Pike G. B. Siddiqi K. (2006). 3D curve inference for diffusion MRI regularization and fibre tractography. *Med. Image Anal.* 10 799–813. 10.1016/j.media.2006.06.009 16919994

[B102] Schleicher A. Amunts K. Geyer S. Morosan P. Zilles K. (1999). Observer-independent method for microstructural parcellation of cerebral cortex: a quantitative approach to cytoarchitectonics. *Neuroimage* 9 165–177. 10.1006/nimg.1998.0385 9918738

[B103] Seehaus A. K. Roebroeck A. Chiry O. Kim D. S. Ronen I. Bratzke H. (2013). Histological validation of DW-MRI tractography in human postmortem tissue. *Cereb. Cortex* 23 442–450. 10.1093/cercor/bhs036 22345356 PMC3584953

[B104] Sherbondy A. J. Dougherty R. F. Ananthanarayanan R. Modha D. S. Wandell B. A. (2009). Think global, act local; projectome estimation with BlueMatter. *Med. Image Comput. Comput. Assist. Interv.* 12 861–868. 10.1007/978-3-642-04268-3_106 20426069 PMC3076280

[B105] Sherbondy A. J. Dougherty R. F. Ben-Shachar M. Napel S. Wandell B. A. (2008). ConTrack: finding the most likely pathways between brain regions using diffusion tractography. *J. Vis.* 8 15 1–16. 10.1167/8.9.15 PMC2696074 18831651

[B106] Sotiropoulos S. N. Bai L. Morgan P. S. Constantinescu C. S. Tench C. R. (2010). Brain tractography using Q-ball imaging and graph theory: improved connectivities through fibre crossings via a model-based approach. *Neuroimage* 49 2444–2456. 10.1016/j.neuroimage.2009.10.001 19818861

[B107] Sotiropoulos S. N. Jbabdi S. Xu J. Andersson J. L. Moeller S. Auerbach E. J. (2013). Advances in diffusion MRI acquisition and processing in the Human Connectome Project. *Neuroimage* 80 125–143. 10.1016/j.neuroimage.2013.05.057 23702418 PMC3720790

[B108] Sporns O. Tononi G. Kotter R. (2005). The human connectome: a structural description of the human brain. *PLoS Comput. Biol.* 1:e42 10.1371/journal.pcbi.0010042 PMC1239902 16201007

[B109] Stanisz G. J. Szafer A. Wright G. A. Henkelman R. M. (1997). An analytical model of restricted diffusion in bovine optic nerve. *Magn. Reson. Med.* 37 103–111. 10.1002/mrm.1910370115 8978638

[B110] Stejskal E. O. Tanner J. E. (1965). Spin diffusion measurements: spin echoes in the presence of a time-dependent field gradient. *J. Chem. Phys.* 42 288–292. 10.1063/1.1695690

[B111] Stephan K. E. Zilles K. Kotter R. (2000). Coordinate-independent mapping of structural and functional data by objective relational transformation (ORT). *Philos. Trans. R. Soc. Lond. B Biol. Sci.* 355 37–54. 10.1098/rstb.2000.0548 10703043 PMC1692724

[B112] Stikov N. Campbell J. Boudreau M. Narayanan S. Stroh T. Nuara S. (2014). “In vivo histology of the myelin g-ratio,” in *Proceedings of the 2014 Organization for Human Brain Mapping Annual Meeting* Hamburg 2249.

[B113] Talairach J. Tournoux P. (1988). *Co-Planar Stereotaxic Atlas of the Human Brain : 3-Dimensional Proportional System : An Approach to Cerebral Imaging*. Stuttgart: Georg Thieme.

[B114] Thomas C. Ye F. Q. Irfanoglu M. O. Modi P. Saleem K. S. Leopold D. A. (2014). Anatomical accuracy of brain connections derived from diffusion MRI tractography is inherently limited. *Proc. Natl. Acad. Sci. U.S.A.* 111 16574–16579. 10.1073/pnas.1405672111 25368179 PMC4246325

[B115] Tomassini V. Jbabdi S. Klein J. C. Behrens T. E. Pozzilli C. Matthews P. M. (2007). Diffusion-weighted imaging tractography-based parcellation of the human lateral premotor cortex identifies dorsal and ventral subregions with anatomical and functional specializations. *J. Neurosci.* 27 10259–10269. 10.1523/JNEUROSCI.2144-07.2007 17881532 PMC6672665

[B116] Tournier J. D. Calamante F. Connelly A. (2007). Robust determination of the fibre orientation distribution in diffusion MRI: non-negativity constrained super-resolved spherical deconvolution. *Neuroimage* 35 1459–1472. 10.1016/j.neuroimage.2007.02.016 17379540

[B117] Tournier J. D. Calamante F. Connelly A. (2010). “Improved probabilistic streamlines tractography by 2nd order integration over fibre orientation distributions,” in *Proceedings of the Joint Annual Meeting ISMRM-ESMRMB 2010* Stockholm.

[B118] Tournier J. D. Calamante F. Gadian D. G. Connelly A. (2004). Direct estimation of the fiber orientation density function from diffusion-weighted MRI data using spherical deconvolution. *Neuroimage* 23 1176–1185. 10.1016/j.neuroimage.2004.07.037 15528117

[B119] Tuch D. S. (2004). Q-ball imaging. *Magn. Reson. Med.* 52 1358–1372. 10.1002/mrm.20279 15562495

[B120] Tuch D. S. Reese T. G. Wiegell M. R. Makris N. Belliveau J. W. Wedeen V. J. (2002). High angular resolution diffusion imaging reveals intravoxel white matter fiber heterogeneity. *Magn. Reson. Med.* 48 577–582. 10.1002/mrm.10268 12353272

[B121] van den Heuvel M. P. Mandl R. C. Stam C. J. Kahn R. S. Hulshoff Pol H. E. (2010). Aberrant frontal and temporal complex network structure in schizophrenia: a graph theoretical analysis. *J. Neurosci.* 30 15915–15926. 10.1523/JNEUROSCI.2874-10.2010 21106830 PMC6633761

[B122] van den Heuvel M. P. Sporns O. (2011). Rich-club organization of the human connectome. *J. Neurosci.* 31 15775–15786. 10.1523/JNEUROSCI.3539-11.2011 22049421 PMC6623027

[B123] Van Essen D. C. Ugurbil K. (2012). The future of the human connectome. *Neuroimage* 62 1299–1310. 10.1016/j.neuroimage.2012.01.032 22245355 PMC3350760

[B124] Vogt O. (1910). Die myeloarchitektonische felderung des menschlichen stirnhirns. *J. Psychol. Neurol.* 15 221–232.

[B125] von Economo C. Koskinas G. N. (1925). *Die Cytoarchitektonik der Hirnrinde des Erwachsenen Menschen*. Vienna: Julius Springer.

[B126] Wakana S. Jiang H. Nagae-Poetscher L. M. Van Zijl P. C. Mori S. (2004). Fiber tract-based atlas of human white matter anatomy. *Radiology* 230 77–87. 10.1148/radiol.2301021640 14645885

[B127] Wang H. Black A. J. Zhu J. Stigen T. W. Al-Qaisi M. K. Netoff T. I. (2011). Reconstructing micrometer-scale fiber pathways in the brain: multi-contrast optical coherence tomography based tractography. *Neuroimage* 58 984–992. 10.1016/j.neuroimage.2011.07.005 21771662 PMC3178460

[B128] Wedeen V. J. Hagmann P. Tseng W. Y. Reese T. G. Weisskoff R. M. (2005). Mapping complex tissue architecture with diffusion spectrum magnetic resonance imaging. *Magn. Reson. Med.* 54 1377–1386. 10.1002/mrm.20642 16247738

[B129] Yeh F. C. Wedeen V. J. Tseng W. Y. (2010). Generalized q-sampling imaging. *IEEE Trans. Med. Imaging* 29 1626–1635. 10.1109/TMI.2010.2045126 20304721

[B130] Zalesky A. Fornito A. (2009). A DTI-derived measure of cortico-cortical connectivity. *IEEE Trans. Med. Imaging* 28 1023–1036. 10.1109/TMI.2008.2012113 19150781

[B131] Zatorre R. J. Fields R. D. Johansen-Berg H. (2012). Plasticity in gray and white: neuroimaging changes in brain structure during learning. *Nat. Neurosci.* 15 528–536. 10.1038/nn.3045 22426254 PMC3660656

[B132] Zhang H. Schneider T. Wheeler-Kingshott C. A. Alexander D. C. (2012). NODDI: practical in vivo neurite orientation dispersion and density imaging of the human brain. *Neuroimage* 61 1000–1016. 10.1016/j.neuroimage.2012.03.072 22484410

[B133] Zikopoulos B. Barbas H. (2011). “Comparable organization of frontal white matter in human and non-human primates,” in *Proceedings of the 2011 Society for Neuroscience Annual Meeting* Washington, DC: Society for Neuroscience.

[B134] Zilles K. Amunts K. (2009). Receptor mapping: architecture of the human cerebral cortex. *Curr. Opin. Neurol.* 22 331–339. 10.1097/WCO.0b013e32832d95db 19512925

[B135] Zilles K. Amunts K. (2010). TIMELINE centenary of Brodmann’s map – conception and fate. *Nat. Rev. Neurosci.* 11 139–145. 10.1038/nrn2776 20046193

